# Jiawei Yanghe Decoction Regulates Bone-Lipid Balance through the BMP-SMAD Signaling Pathway to Promote Osteogenic Differentiation of Bone Mesenchymal Stem Cells

**DOI:** 10.1155/2022/2885419

**Published:** 2022-06-20

**Authors:** Yunfeng Luo, Hanting Xia, Jiacai Wang, Qian Hu, Yinghua Luo, Jiangyuan Liu, Zhijun Yang, Wei Li, Hongyu Wang, Fuwei Li, Zhaochong Mao, Wenlong Yang, Fengyun Yang

**Affiliations:** ^1^Graduate School, Jiangxi University of Chinese Medicine, Nanchang 330000, Jiangxi, China; ^2^Department of Orthopedics, Affiliated Hospital of Jiangxi University of Chinese Medicine, Nanchang 330000, Jiangxi, China; ^3^Institute of Orthopaedics and Traumatology, The First Affiliated Hospital of Zhejiang Chinese Medical University, Hangzhou 310005, Zhejiang, China; ^4^Department of Internal Medicine, Nanchang County Hospital of Traditional Chinese Medicine, Nanchang 330000, Jiangxi, China; ^5^Department of Acupuncture and Moxibustion, Affiliated Hospital of Jiangxi University of Chinese Medicine, Nanchang 330000, Jiangxi, China; ^6^Department of Orthopedics, Shenzhen Pingle Orthopaedic Hospital, Shenzhen 518038, Guangdong, China

## Abstract

**Background:**

The Jiawei Yanghe decoction (JWYHD) is a traditional Chinese medicine formula for the treatment of osteoporosis, but its therapeutic mechanism has not been fully elucidated, and the therapeutic target of the intervention disease needs to be further verified. The dysfunction of bone mesenchymal stem cells (BMSCs) is considered to be an important pathogenesis of postmenopausal osteoporosis (PMOP). The purpose of this study was to explore how JWYHD regulates BMSC differentiation through the BMP-SMAD signal pathway.

**Methods:**

In the in vivo study, we used an ovariectomized PMOP rat (*n* = 36, 2-month-old, 200 ± 20 g) model and femur micro-CT analysis to study the effect of JWYHD on bone loss in rats. By immunofluorescence, the translocation expression of BMP2, a key protein in the pathway, was detected. Serum bone metabolism was detected by an enzyme-linked immunosorbent assay (ELISA). Alkaline phosphatase (ALP) activity was detected by alkaline phosphatase staining (ALPS), osteogenesis and matrix mineralization were detected by alizarin red staining (ARS), the adipogenic ability of BMSCs was detected by oil red staining (ORS), and CFU is used to detect the ability of cells to form colonies. The expression of related proteins was detected by western blotting.

**Results:**

In vivo and in vitro, the OP phenotypes of SD rats induced by ovariectomy (OVX) included impaired bone mineral density and microstructure, abnormal bone metabolism, and impaired MSC differentiation potential. JWYHD treatment reversed this trend and restored the differentiation potential of MSCs. JWYHD medicated serum and direct intervention of drugs activated the BMP-SMAD signaling pathway, promoted the osteogenic differentiation of BMSCs, and inhibited their adipogenic differentiation.

**Conclusions:**

Our data identified that JWYHD is an effective alternative drug for the treatment of PMOP that functions to stimulate the differentiation of BMSCs into osteoblasts in the BMP-SMAD signaling-dependent mechanism.

## 1. Introduction

Postmenopausal osteoporosis (PMOP) is a disease of bone metabolism associated with estrogen deficiency due to cessation of ovarian function, occurring mainly in postmenopausal women and characterized by changes in bone microarchitecture and reduced bone mass [1, 2]. From 2008 to 2018, according to relevant epidemiological reports, the prevalence of osteoporosis is 6.46% in elderly men and 29.13% in women [3]. The prevalence of osteoporotic fractures is increasing at an alarming rate globally, with 6.7% of people over 50 years of age experiencing nontraumatic fractures [4], with more than 75% of osteoporotic fracture patients being women, and is a major cause of death and disability, creating a significant public health burden worldwide [5, 6].

Bone mesenchymal stem cells (BMSCs) are adult stem cells existing in the bone marrow matrix, which can further differentiate into osteoblasts, adipocytes, chondrocytes, muscle cells, etc [7, 8]. It has been found that bone differentiation and lipid differentiation restrict each other, and the imbalance of either side may lead to osteoporosis [9, 10]. Furthermore, clinical studies have revealed that the number of trabeculae in the bone marrow is inversely proportional to the amount of adipose tissue [11]. Therefore, enhancing osteogenesis and reducing adipogenesis can increase bone formation by shifting the adipogenesis of BMSCs to osteogenesis. BMSCs have become a hot target for the prevention and treatment of osteoporosis because of the multidirectional differentiation potential, especially the ability to differentiate into osteogenesis [12–14].

The BMP-SMAD (bone morphogenetic protein-Drosophila mothers against decapentaplegic) signaling pathway is not only a critical pathway for regulating the differentiation and maturation of BMSCs into osteoblasts, but also one of the most critical factors in regulating bone development and bone metabolism [15–17]. BMPs are abundant in the bone matrix and have been found to induce ectopic osteogenesis in muscle [18, 19]. Research has confirmed that the BMP-SMAD signaling pathway might regulate all aspects of the osteoblast life cycle, including BMSC differentiation into osteoblasts, osteoblast expansion, osteoblast mineralization, and coupling with osteoclasts [20]. For example, loss of the BMP signaling pathway can lead to osteoporosis, which is accompanied by defects in osteogenic differentiation. The BMP knockout mice exhibit abnormal bone development and defects in osteoblast function [21]. The mice with BMPRIA (BMP receptor IA) deletion in osteoblasts [22] or overexpression of Noggin (inhibitor of BMP signaling pathway) [23] had osteopenia, a lower number of osteoblasts, and defects in differentiation and mineralization. Previous studies have shown that BMPs enhance the function of osteoblasts by promoting collagen synthesis and mineral deposition [24]. This pathway is a major hotspot in the research on the pathogenesis of PMOP at the level of molecular biology.

Jiawei Yanghe decoction (JWYHD) originated in the Qing Dynasty hundreds of years ago. Wang Hongxu, a famous Chinese medical doctor, first recorded Yanghe Decoction in his work (*Waike Zhengzhi Quansheng Ji*), and it has proved to be useful in the treatment of osteoporosis and osteoarthritis. In recent years, JWYHD has gradually developed into a clinically famous formula that has the functions of benefiting bone marrow, tonifying the liver and kidney, promoting blood circulation, and dredging collaterals. Traditional Chinese medicine believes that osteoporosis is bone impotence, which is closely related to kidney function, so a deficiency of kidney essence and an empty marrow can easily lead to osteoporosis. The treatment is mainly to tonify the kidney essence and benefit the bone marrow. As a traditional Chinese medicine formula for the treatment of osteoporosis, JWYHD is widely used in clinics because of its reliable curative effects and nontoxic side effects. Previous studies have shown that it might protect the extracellular matrix of cartilage, improve the activity of osteoblasts, reduce bone metabolism, and improve bone microstructure [25]. JWYHD is a kind of Bushen Huoxue decoction. Studies have shown that Bushen Huoxue decoction can suppress the experimental autoimmune thyroiditis in rats by improving NLRP3 inflammasome and immune dysregulation [26], antagonize the decline of estrogen in ovarian rats [27], and enhance the estrogen-progesterone signal axis [28]. Some studies have shown that *Rehmannia glutinosa* activates mesenchymal progenitor cells through the TGF-*β* signal to promote fracture healing [29]. At the same time, *Rehmannia glutinosa* (the dried tuberous root of Scrophulariaceae plants is considered by traditional Chinese medicine to have the functions of enriching the blood, tonifying the kidneys, and filling the marrow) extract could improve the ALP activity and osteogenic differentiation ability of embryonic osteogenic progenitor cells through the IGF-1/PI3K/mTOR pathway and prevent bone loss in rats [30]. It was discovered that a fermented papaya preparation might lengthen bone marrow telomeres and boost antioxidation levels, which played a key role in redox balance and prevention of age-related molecular damage diseases in a mouse model [31]. Papaya inhibited the differentiation and function of osteoporosis induced by RANKL and effectively improved the osteoporosis induced by OVX in rats [32]. Cinnamon extract has an estrogenic effect, which might increase the content of serum E2 and collagen 1, significantly reduce the loss of bone mineral and bone mass of femur in OVX rats, and inhibit body weight gain [33]. Tetrandrine (the dried root tuber of Menispermaceae is considered by traditional Chinese medicine to have the effect of dispelling rheumatism, promoting diuresis, and relieving swelling) could reduce the level of intracellular oxidative stress against osteoporosis through the TGF-*β*1/Nrf-2/HO-1 signal pathway [34].

Over the years, modern medicine has isolated bioactive and medicinally related compounds from traditional Chinese medicine, and numerous new drugs have been discovered. Traditional Chinese medicine prescriptions composed of two or more herbs usually have better curative effects and fewer side effects than those composed of single herbs. In recent years, several lines of evidence show that due to the complex pathogenesis and progress of diseases, a single active drug ingredient or Chinese herbal medicine can only produce moderate efficacy, and these diseases often lead to a variety of side effects or drug resistance. Because of its multicomponent, multi-target, and synergistic characteristics, traditional Chinese medicine has been proven to be very suitable for the treatment of complex diseases and their symptoms [35]. Traditional Chinese medicine, as an important part of complementary and alternative medicine, has developed into an attractive source of multi-target drugs and has played an important role in the prevention and treatment of various diseases [36].

In this study, we explored how JWYHD regulates the differentiation of BMSCs through the BMP-SMAD signaling pathway to combat the pathological process of osteoporosis. Its effects on osteogenesis and adipogenic differentiation of BMSCs in OVX rats were evaluated in vitro and in vivo. This information may help to clarify the role of JWYHD in bone metabolism and its mechanism against osteoporosis.

## 2. Materials and Methods

### 2.1. Composition and Preparation of JWYHD Drugs

The Chinese herbal medicines used in JWYHD are shown in Table 1. All crude herbs were soaked in 5 times the volume of water and boiled for 1 hour, followed by filtration 3 times. The resultant decoction was concentrated until the concentration of the drug was set at 1.5 g/ml (used for in vivo experiments). Part of the original decoction continued drying to obtain decoction powder. Dry extraction was achieved using DMSO at a concentration of 1 g/ml and stored at −20°C (used for in vitro experiments). The clinical dose was calculated according to the ratio of human to mouse (body weight 60 kg).

### 2.2. Animal Research

All-female SD mice (*n* = 36, 2-month-old, 200 ± 20 g) were obtained from the Animal Experimental Center of Jiangxi University of Traditional Chinese Medicine. All rats were free to access diet and water. The Committee for the Management and Use of Experimental Animals of Jiangxi University of Traditional Chinese Medicine approved all animal experiments and studies (the ethical approval number: JZLLSC20220012). All animal experiments comply with the National Institutes of Health's guidelines for the care and use of experimental animals.

The rat PMOP model was established by bilateral ovariectomy [37, 38]. The anesthesia method was initiated with an intraperitoneal injection of pentobarbital, and then the rats were fixed in a supine position for surgery. The surgical area was disinfected, towels were laid, the uterus was positioned, and a longitudinal incision of about 1 cm was made in the abdomen. In the sham group, both ovaries were retained, and only the fat with similar weight and volume (weight: 70 mg, volume: 5∗4∗3 mm^3^) around the ovaries was removed, while the other groups had both ovaries removed. All rats were randomly divided into 6 groups: (1) the control group (SHAM), (2) the bilateral ovaries resection group (OVX), (3) the OVX + *β*-estradiol (500 ug/kg/d) in the treatment group (E2), (4) the OVX + JWYHD (0.75 g/kg/d) low-dose treatment groups (L), (5) OVX + JWYHD (1.5 g/kg/d) medium-dose treatment groups (M), and (6) the OVX + JWYHD (3.0 g/kg/d) high-dose treatment group (H).

After 12 weeks of drug treatment, blood samples and femur tissues were collected for further analysis. The left femur of each group was scanned by micro-CT, and the right femur and tibia of each group were collected to obtain BMSCs, which were cultured for ARS, ALPS, ORS, WB analysis, and CFU assay.

### 2.3. Analysis of Chemical Composition of JWYHD Medicated Serum by LC-MS

The samples were analyzed by LC-MS, and the chromatographic conditions were as follows: Chromatographic column: AQ-C18, 150 × 2.1 mm, 1.8 um, Welch; flow rate: 0.30 ml/min; aqueous phase: aqueous solution of 0.1% formic acid; organic phase: methanol; needle washing liquid: methanol; column temperature: 35°C; automatic sampler temperature: 10.0°C; and automatic sampler injection volume: 5.00 *μ*L. The mobile phases were composed of an aqueous solution of 0.1% formic acid (A) and methanol (B). The gradient elution program was (time/B%): 0−1 min, 2%; 1−5 min, 2%–20%; 5−10 min, 20–50%; 10−15 min, 50–80%; 15–20 min, 80–95%; 20−25 min, 95%; and 26–30 min, 2%. The conditions of mass spectrometry are follows: ion source: electrospray ionization source; scanning mode: positive and negative ion switching scanning; detection method: full mass/dd-MS2; resolution: 70000 (full mass), 17500 (dd-MS2); scanning range: 150.0 mm-2000.0 m/z; electrospray voltage: 3.8 kV (positive); capillary temperature: 300°C; collision gas: high purity argon (purity ≥99.999%); sheath gas: nitrogen (purity ≥99.999%), 40 Arb; auxiliary gas: nitrogen (purity ≥99.999%), 350°C; and data acquisition time: 30.0 min. In the SHAM group, 2 hours after the last treatment, blood samples from the abdominal aorta were collected to prepare drug-containing serum. Then, the serum sample was centrifuged at 8000 × *g* for 5 min, and 2 ml was added to 3 times the volume of methanol. After rotating for 15 min, the supernatant of the sample was volatilized to dry under vacuum, then dissolved with 100 ml of methanol, and centrifuged for 15 min at 12000 rpm/min. Then, the sample was filtered through the microporous membrane (0.22 *μ*m) to obtain the drug-containing serum sample. The data collected by a high-resolution mass spectrometer were preliminarily sorted out by CD2.1 (ThermoFisher) and then compared with the database (mzCloud, mzVault, ChemSpider).

### 2.4. Isolation and Culture of BMSCs

The rat BMSCs used in this study were obtained from the femur and tibia of rats. The connective tissue of the femur and tibia was removed, and after cutting both ends of the bone, the bone marrow was flushed out of the femur and tibia with a syringe suction medium consisting of a-mem (Icell, China, #1480012), 20% FBS (Gibco, USA, #10099–141), 1% penicillin-streptomycin, and L-glutamine (Invitrogen, USA, #D16703) 12 mM. Then the flushed cell suspension was cultured in a culture flask, and the culture medium was changed every 2–3 days. At 100% confluence, the cells were digested with 0.25% trypsin containing 1 mM EDTA (Sigma-Aldrich, USA, #93615) and subcultured until the third generation for further experiments.

### 2.5. Cell Proliferation Assay

The proliferation was detected by the MTT (3-(4,5)-dimethylthiahiazo (-z-y1)-3,5-di- phenytetrazoliumromide) assay. The cells were inoculated into a 96-well plate with an initial density of 1 × 10^3^ per well. After 12 hours of serum starvation, JWYHD-containing serum or JWYHD drugs were added to the culture medium. 20 *μ*l of 5%MTT solution (Solarbio, China, #M1020) was added to the culture medium at 24 h and 48 h, respectively. The supernatant was removed, 150 *μ*l DMSO solution (Solarbio, China, #D8370) was added, and the absorbance was measured at 570 nm wavelength.

### 2.6. ELISA

In vivo, the plasma samples of rats in each group were centrifuged at a rotational speed of 1.5 × 10^4^ for 10 minutes, and then the supernatant was collected. The corresponding ELISA kit was used to detect the levels of bone formation biochemical markers P1NP (Elabscience, China, #E-EL-R1414c) and bone resorption biochemical markers *β*-CTX (Elabscience, China, #E-EL-R1405c) according to the instructions of the manufacturer.

### 2.7. Western Blotting Analysis

The cells were treated with RIPA cleavage buffer containing phosphatase and protease inhibitor (Sigma-Aldrich, USA, #89900). The protein concentration was quantified by the BCA protein detection kit (ThermoFisher, USA, #23225). The protein was separated by 10% SDS-polyacrylamide gel electrophoresis and then transferred to the PVDF membrane. The imprinted membrane was blocked by TBST containing 5% BSA for 1 h. The first antibody against BMP2 (Abcam, UK, #ab214821), P-SMAD1/5/8 (Santa Cruz, USA, #sc-12353P), RUNX2 (Abcam, UK, #ab236639), OCN (Abcam, UK, #ab133612), PPAR*γ*(Abcam, UK, #ab178860), and *β*-actin (Abcam, UK, #ab8226) was incubated at 4°C for 14 h, and then the membrane was washed and incubated with goat anti-rabbit (ThermoFisher, USA, #A21076) or goat anti-mouse (ThermoFisher, USA, #A21094) secondary antibody at room temperature for 1 h. The protein was observed by the ECL Western blotting imaging system (ThermoFisher, USA, #32209). The Image Lab software (Bio-Rad, USA) was used to quantify the gray value of protein bands.

### 2.8. Determination of ALP Activity

The ALPS was used to evaluate the activity of ALP after osteogenic differentiation of BMSCs according to the method of the BCIP/NBT staining kit (Beyotime, China, #C3206), removed the culture medium, washed it twice with precooled PBS, added 4% paraformaldehyde fixing solution, placed it at room temperature for 10 minutes, washed it twice, added substrate solution to each well, and incubated it in an incubator at 37°C for 45 minutes. During this time, it was vital to observe constantly, avoid over staining, and remove the solution. After being washed, cells were observed using a Leica Confocal 222 microscope. ImageJ software (NCI, USA) was used to analyze the images.

### 2.9. BMSCs Induced Differentiation into Bone Pedigree

The extracted BMSCs of each group were seeded at a 6-well plate containing the growth medium at a density of 1 × 10^4^ per well. After the cell culture reached 70%–80% confluence, the growth medium was switched to an osteogenesis inducing solution: *α*-MEM, 0–15% serum, 10 mM sodium *β*-glycerophosphate (Solarbio, China, #G81100), 50 *μ*g/mL ascorbic acid (Sigma-Aldrich, USA, #255564), and 10 nM dexamethasone (Sigma-Aldrich, USA, #D1756). The inducing solution was changed every 3 days, and after 21 days, it was treated with 4% paraformaldehyde cell fixative for 15 minutes, and then stained with ARS (Sigma-Aldrich, USA, #A5533) for 30 minutes. After being washed, cells were observed using a Leica Confocal 222 microscope. ImageJ software was used to analyze the images.

### 2.10. BMSCs Induced Differentiation into Lipid Lineage

ORS was used to analyze lipid formation. The growth medium was transferred to the adipogenic induction medium: DMEM, 0–15% serum, 1% penicillin-streptomycin, 200 *μ*M indomethacin (Sigma-Aldrich, USA, #17378), 1 *μ*M dexamethasone (Sigma-Aldrich, USA, #D1756), 10 *μ*g/mL insulin (Sigma-Aldrich, USA, #12643), and 0.5 mM 3-Isobutyl-1-methylxanthine (IBMX, Sigma-Aldrich, USA, #15879) after 2 days of induction and cultured for 5 days in adipogenic maintenance medium (DMEM, 10% FBS, 10 g/mL insulin). The culture medium was changed every 2 days. The cells were fixed with 4% paraformaldehyde for 15 minutes after 7 days. Then, the cells were stained with 1 ml/well oil red staining solution (Solarbio, China, #G1262) at room temperature for 20 minutes. After being washed, cells were observed using a Leica Confocal 222 microscope. ImageJ software was used to analyze the images.

### 2.11. CFU Analysis

To evaluate the colony-forming ability of BMSCs, each group was seeded at a 6-well plate with a cell density of 1 × 10^4^ per well. After 7 days, the cells were treated with cell fixation solution for 10 minutes, then stained with 0.1% toluidine blue (Solarbio, China, #G3660) for 20 minutes at room temperature, washed, and air-dried. ImageJ software was used to analyze the images.

### 2.12. Micro-CT and Bone Histomorphometric Analysis

The femur was scanned and remodeled by micro-CT (Skyscan 1176, Belgium). For the distal femur, we selected the region of interest (ROI) 1.5 mm–4.0 mm above the growth plate for analysis. The distal end of the right femur was scanned, and the bone mass of the femur was quantitatively analyzed, including bone mineral density (BMD), trabecular volume (BV/TV), trabecular thickness (Tb.Th), trabecular number (Tb.N), and trabecular separation (Tb.Sp). The results were analyzed by *μ*CT version 1.1.1 software.

### 2.13. Immunofluorescence

The tissue sections to be dyed were baked in an oven at 65 degree Celsius for 1 hour and then dewaxed. PBS washed 3 times for 5 minutes each time. A citrate buffer solution was used for antigen repair. In order to achieve good results, the microwave oven was first microwaved at high heat for 3 minutes and then converted to low heat for 15 minutes. For 30 min, it was incubated with 3% H_2_O_2_ at room temperature for the purpose of inactivating endogenous peroxidase. 1% BSA was used to block for 30 min at room temperature to block nonspecific epitopes. Overnight incubation with the specific first antibody, BMP2, was performed in a wet box at 4 °C. The slices were taken out the next day and, for 30 minutes, they were reheated at room temperature. The tissue was incubated for 30 minutes at 37°C with the corresponding immunofluorescent secondary antibody. Under the condition of avoiding light, DAPI staining solution was used to dye the nucleus, and the concentration and time were used according to the reagent instructions. The anti-fluorescence quenching agent was added to the tissue to seal the film. Then, it was observed and photographed by the Leica Confocal 222 Microscope.

### 2.14. Statistical Analysis

GraphPad Prism 8 software was used for data analysis. Unless otherwise specified, the data are representative of at least three experiments with similar results that were performed in triplicate and expressed as mean ± SD. The Shapiro–Wilk test was used to determine the normal distribution of the data, and each group of data obeyed the normal distribution. One-way ANOVA followed by Student–Newman–Keuls post hoc test was used to determine the significant difference. *P* < 0.05 was considered statistically significant.

## 3. Results

### 3.1. Analysis and Identification of JWYHD Drug-Containing Serum by LC-MS

**Figure 1:**
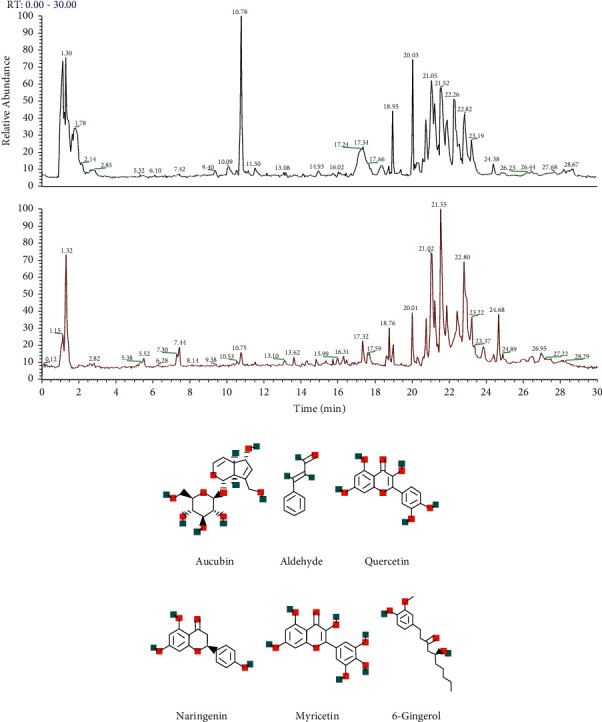
LC-MS was used to analyze and identify the drug-containing serum. (a) Black is the total ion flow diagram of the negative ion mode, and red is the total ion flow diagram of the positive ion mode. (b)-(g) Six compounds were identified from drug-containing serum. PubChem (https://pubchem.ncbi.nlm.nih.gov/) chemical structures of six compounds.

In this study, the prepared drug-containing serum was analyzed by LC-MS. Several main chemical constituents, including aucubin, cinnamaldehyde, quercetin, naringin, myricetin, and 6-gingerol, were identified by LC-MS (Figure 1) and their fingerprints. Through analysis, the contents of six main chemical components were obtained, and the results are shown in Table 2.

### 3.2. JWYHD Could Improve BMSC Dysfunction in Ovariectomized PMOP Rats

**Figure 2:**
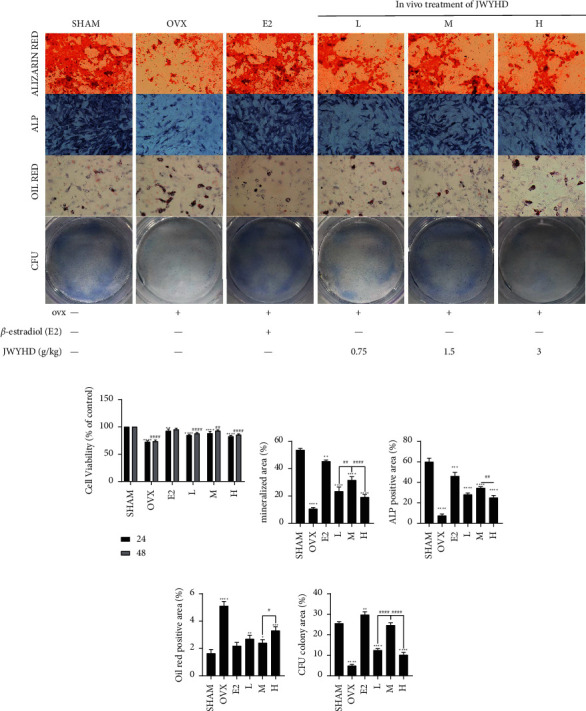
After 12 weeks of JWYHD treatment, the primary BMSCs of each group (SHAM, OVX, E2, L, M, and H were taken to detect the phenotype of the cells. (a) ARS (Scale bar: 200 *μ*m), ALPS (Scale bar: 200 *μ*m), ORS (Scale bar: 200 *μ*m), and CFU (Magnification: 1×). (b) The increment of primary BMSCs at 24 hours and 48 hours. (c) The matrix mineralization area of ARS. (d) The area of ALPS. (e) The lipidation area of ORS. (f) The area of CFU. The data were represented by mean ± SD (*n* = 3), *P* < 0.05 were considered as significant difference: ^∗^ VS. CTRL group, ^#^VS. M group.

To evaluate the functional expression of BMSCs, we extracted the primary cells of each group and performed ARS, ALPS, ORS, and CFU (Figure 2(a)). Phenotypic biological analysis showed that the osteogenic differentiation ability of the OVX group was significantly lower than that of the SHAM group (Figures 2(c) and 2(d)). The CFU assay showed that the self-renewal ability of the OVX group was significantly lower than that of the SHAM group (Figure 2(f)), while ORS showed the opposite result: the lipid differentiation ability of the OVX group was significantly higher than that of the SHAM group (Figure 2(e)). In the MTT cell proliferation assay, the E2 group was used as the positive drug control group, and the results showed that the medium-dose group (M) of JWYHD had the optimal effect on promoting cell proliferation (Figure 2(b)). The above results suggest that JWYHD could improve the dysfunction of BMSCs and restore the potential of osteogenic differentiation in ovariectomized PMOP rats.

### 3.3. JWYHD Attenuated Bone Loss in OVX Rats and Partially Reversed the Pathological Process of PMOP

**Figure 3:**
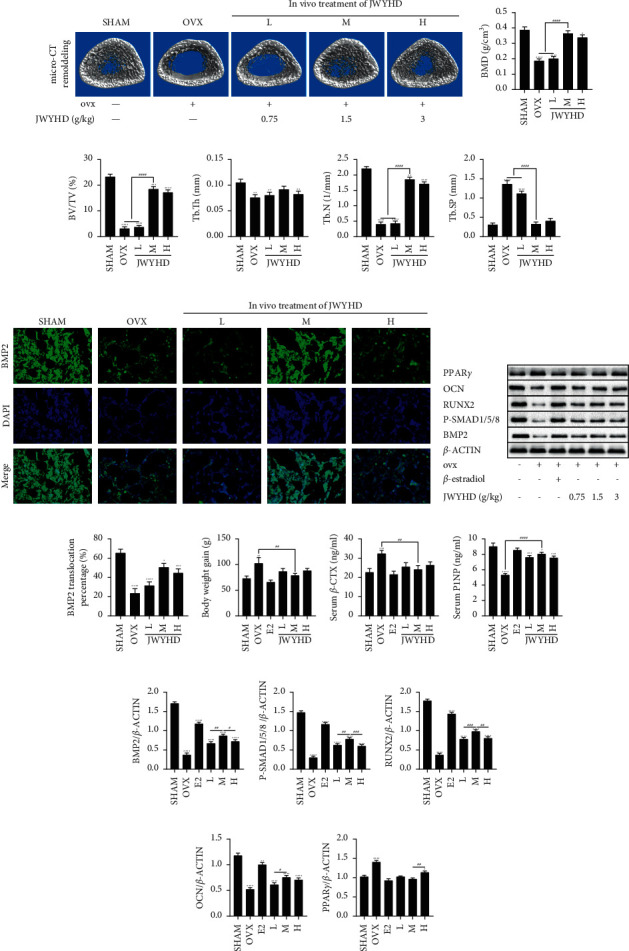
The changes in bone metabolism parameters and serological markers in rats after 12 weeks of JWYHD intragastric treatment are described. (a) Micro-CT was used to scan three-dimensional reconstruction images of bone trabeculae. (b) BMD. (c) BV/TV. (d) Tb.Th. (e) Tb.N. (f) Tb.Sp. (g) The translocation expression of BMP2, a key protein in the pathway, was detected by immunofluorescence. (h) The protein expression was measured by WB after culturing for 1 week. (i) BMP2 translocation percentage. (j) Weight gain of rats. (k) ELISA quantified the content of bone resorption marker *β*-CTX in serum. (l) ELISA quantified the content of the bone formation marker P1NP in serum. (m)-(q) The quantitative measurement of protein expression (BMP2, P-SMAD1/5/8, RUNX2, OCN, PPAR*γ*). The data were represented by mean ± SD (*n* = 3), *P* < 0.05 were considered as significant difference: ^∗^ VS. SHAM group, ^#^VS. M group.

After 12 weeks of drug treatment, micro-CT scanning and three-dimensional reconstruction were used to quantitatively analyze the bone parameters of rats in each group (SHAM, OVX, *L*, M, and H) (Figure 3(a)). The results of micro-CT data analysis showed that compared with the control group, the bone parameters BMD, BV/TV, Tb.N, and Tb.Th in other groups decreased significantly (Figures 3(b)–3(e)), while Tb.Sp increased (Figure 3(f)). These results show that the establishment of the PMOP model induced by OVX in rats was successful. Moreover, groups M and H all showed significant anti-bone mass loss, among which group M (1.5 g/kg/d) had the most significant effect. The BMP2 translocation expression was detected using immunofluorescence (Figure 3(g)), and the percentage of BMP2 translocation expression in the other groups was significantly different from the control group (Figure 3(i)). After 12 weeks of drug treatment, compared with the SHAM group, the weight gain of the OVX group was the most obvious (Figure 3(j)). The serum bone resorption marker serum *β*-CTX significantly increased in the OVX group (Figure 3(k)), indicating that bone resorption in the OVX group was active. The serum bone formation marker serum P1NP in the OVX group was significantly lower than that in the SHAM group (Figure 3(l)), indicating that bone formation was inhibited in the OVX group. After quantification of WB, it was found that JWYHD promoted the protein expression of upstream genes BMP2, P-SMAD1/5/8 (Figures 3(m) and 3(n)), and downstream osteogenesis-related markers (RUNX2, OCN) (Figures 3(o) and 3(p)), while the adipogenesis-related marker PPAR*γ* decreased significantly at protein level (Figure 3(q)). To sum up, in vivo, JWYHD reversed the pathological process of OP induced by OVX, including decreased bone mineral density, bone microstructure damage, BMSC loss, and lipid vacuole formation, suggesting that JWYHD may exert its anti-OP effect by activating the BMP-SMAD signal pathway in BMSCs.

### 3.4. JWYHD-Mediated Osteogenic Differentiation of BMSCs in a Concentration-dependent and Dose-dependent Manner

**Figure 4:**
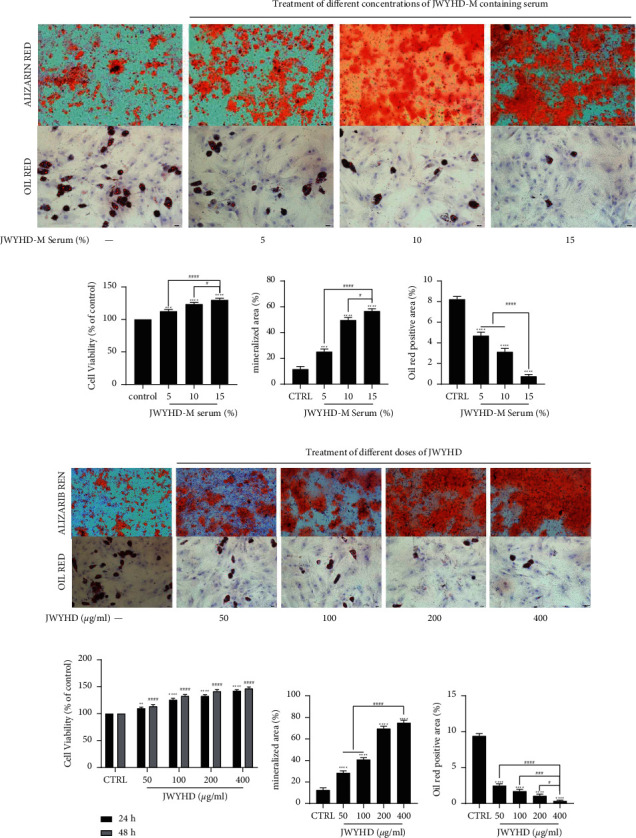
After different concentrations of JWYHD-M containing serum and different doses of JWYHD intervened in the BMSCs, the cell differentiation was detected. (a) ARS and ORS (Scale ‘bar: 200 *μ*m). (b) MTT was used to determine the value-added degree of 48 h BMSCs cultured in serum containing varying concentrations of JWYHD-M (0–15%). (c) The ARS matrix mineralization area. (d) The ORS lipidation area. (e) ARS and ORS (Scale bar: 200 *μ*m). (f) The increase in BMSCs after 24 and 48 hours of treatment with different doses (0–400 *μ*g/ml) of JWYHD. (g) The ARS matrix mineralization area. (h) The ORS lipidation area. The data were expressed as mean ± standard deviation (*n* = 3), *P* < 0.05 were considered as significant difference: ^∗^ VS. CTRL group, ^#^VS. 15% JWYHD-M serum group or 400 *μ*g/ml JWYHD group.

In order to determine the relationship between the optimal concentration and dose of JWYHD in regulating the osteogenic differentiation of BMSCs, we performed an ARS and an ORS assay (Figures 4(a) and 4(e)). The results of cell differentiation induced by drug-containing serum showed that 15% JWYHD-M serum promoted the highest degree of matrix mineralization (Figure 4(c)), while ORS showed the lowest degree of adipogenic differentiation of BMSCs induced by 15% JWYHD-M serum (Figure 4(d)). At the same time, the MTT assay also showed that 15% JWYHD-M serum had the most significant promoting effect on BMSC proliferation (Figure 4(b)). After the direct intervention of JWYHD drugs to induce cell differentiation, quantitative analysis of ARS showed that JWYHD (400 *μ*g/ml) promoted the highest degree of matrix mineralization (Figure 4(g)), while ORS showed that the degree of adipogenic differentiation of BMSCs' induced by JWYHD (400 *μ*g/ml) was the lowest (Figure 4(h)). At the same time, the MTT assay also showed that JWYHD (400 *μ*g/ml) had the most significant promoting effect on BMSCs' proliferation (Figure 4(f)). To sum up, JWYHD significantly promoted the proliferation of BMSCs, increased osteogenic potential, and decreased adipogenic potential in a concentration- and dose-dependent manner. In this study, the direct intervention of serum containing 15% JWYHD-M and 400 *μ*g/ml JWYHD drug had the best effect on the culture of bone marrow mesenchymal stem cells in vitro.

### 3.5. JWYHD Promoted Osteogenic Differentiation of BMSCs by Activating the BMP-SMAD Signal Pathway

**Figure 5:**
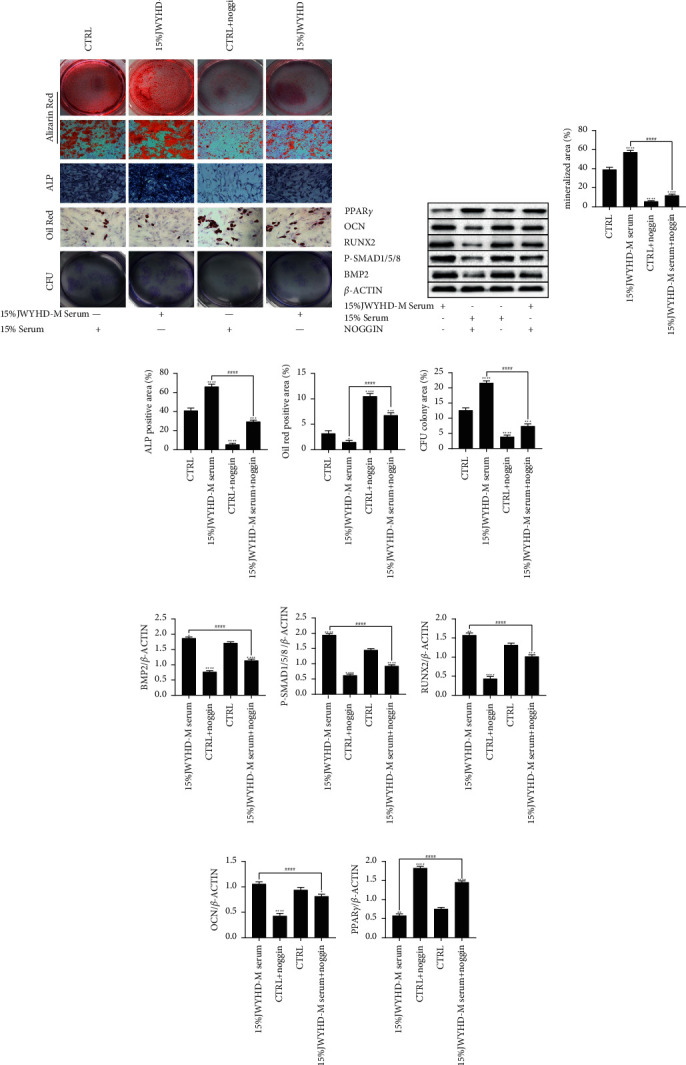
The optimal concentration (15% JWYHD-M serum) intervened in the BMSCs, which were treated with Noggin (100 ng/ml), and both the cell phenotype and protein expression were detected. (a) ARS (Magnification: 1×), ALPS (Scale bar: 200 *μ*m), ORS (Scale bar: 200 *μ*m), and CFU (Magnification: 1×). (b) The expression of osteogenic and adipogenic proteins was measured by WB after culturing for 1 week. (c) The matrix mineralization area of ARS. (d) The area of ALPS. (e) The lipidation area of ORS. (f) The area of CFU. (g)-(k) The quantitative measurement of protein expression (BMP2, P-SMAD1/5/8, RUNX2, OCN, and PPAR*γ*). The data were represented by mean ± SD (*n* = 3), *P* < 0.05 were considered as a significant difference: ^∗^ VS. CTRL group, ^#^VS. 15% JWYHD-M serum group.

**Figure 6:**
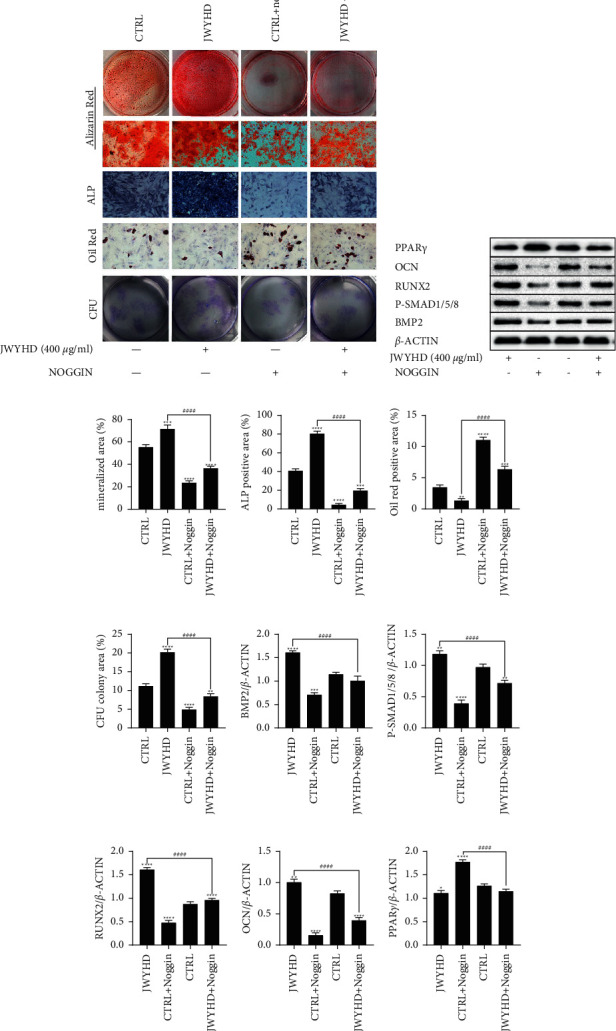
The optimal dose of JWYHD (400 *μ*g/ml) intervened in the BMSCs, which were treated with Noggin (100 ng/ml), and both the cell phenotype and protein expression were detected. (a) ARS (Magnification: 1×), ALPS (Scale bar: 200 *μ*m), ORS (Scale bar: 200 *μ*m), and CFU (Magnification: 1×). (b) The expression of osteogenic and adipogenic proteins was measured by WB after culturing for 1 week. (c) The matrix mineralization area of ARS. (d) The area of ALPS. (e) The lipidation area of ORS. (f) The area of CFU. (g)-(k) The quantitative measurement of protein expression (BMP2, P-SMAD1/5/8, RUNX2, OCN, and PPAR*γ*). The data were represented as mean ± SD (*n* = 3), *P* < 0.05 were considered as a significant difference: ^∗^ VS. CTRL group, ^#^VS. JWYHD (400 *μ*g/ml) group.

To verify that JWYHD promoted osteogenic differentiation of BMSCs by activating the BMP-SMAD signaling pathway, BMSCs were treated with Noggin, and then divided into two groups. Cells were treated with drug intervention and drug-containing serum for ARS, ALPS, ORS, and CFU assay (Figures 5(a) and 6(a)). The results of cell biological characteristic analysis showed that compared with the CTRL group, the bone differentiation ability of the JWYHD group was significantly enhanced, while that of the BMSC group was significantly decreased, and the mineralization degree of the bone differentiation matrix of the JWYHD + noggin group was significantly higher than that of the CTRL + noggin group, and the quantitative analysis results of ARS showed this view (Figures 5(c) and 6(c)). Similarly, the quantitative analysis of ALPS supported this view: compared with the CTRL group, the ALP activity of the JWYHD intervention group was significantly increased, while the BMSC ALP activity of the CTRL + noggin group was significantly decreased, and the ALP activity of the JWYHD + noggin group was significantly higher than that of the CTRL + noggin group (Figures 5(d) and 6(d)). The same results were obtained by quantitative analysis of the CFU assay (Figures 5(f) and 6(f)). The quantitative analysis of ORS showed that the degree of adipogenic differentiation in the JWYHD group was lower than that in the CTRL group, the adipogenic differentiation degree of BMSCs in the CTRL + noggin group was significantly higher than that in the CTRL group, and the adipogenic differentiation degree of the JWYHD + noggin group was significantly lower than that in the CTRL + noggin group (Figure 5(e) and 6(e)). The WB (Figures 5(b) and 6(b)) analysis results showed that compared with the CTRL group, the expression of upstream genes (BMP2, P-SMAD1/5/8) and downstream osteogenesis-related markers (RUNX2, OCN) in the JWYHD group was significantly higher than that in the CTRL + noggin group (Figures 5(g)–5(j) and Figures 6(g)–6(j)), while the protein level in the CTRL + noggin group was significantly decreased, and the expression of bone-related proteins in the JWYHD + noggin group was significantly higher than that in the CTRL + noggin group. However, the expression of PPAR*γ*, a marker related to adipogenesis, was opposite to that of the former at the protein level (Figures 5(k) and 6(k)). To sum up, BMSCs treated with Noggin, an inhibitor of the BMP-SAMD signal pathway, showed a similar impairment of differentiation potential to BMSCs in ovariectomized rats. JWYHD could significantly reverse the dysfunction of BMSCs by activating the BMP-SMAD signal pathway. Both the direct intervention of drugs and the intervention of drug-containing serum showed stable promotion of osteogenic differentiation, and the osteogenic effect was relatively stable.

## 4. Discussion

The purpose of this study is to explore the effect of JWYHD, a traditional Chinese medicine formula with a significant clinical effect on osteoporosis, on the differentiation of BMSCs into osteoblasts and adipocytes in vivo and in vitro. Our data showed that JWYHD could promote the differentiation of BMSCs into osteoblast line and inhibit their differentiation into adipocyte line. This mechanism was mediated by activating the BMP-SMAD signal pathway.

Postmenopausal osteoporosis is a disease of bone metabolism associated with estrogen deficiency due to the cessation of ovarian function. After menopause, the estrogen level of women decreases, the cancellous bone degenerates, and bone mass decreases. At present, the treatment of PMOP is mainly estrogen replacement, calcitonin, bisphosphonates, selective estrogen receptor modulators, and other treatments, but these drugs are toxic and have side effects and are accompanied by adverse reactions, which are controversial, and are not recommended by the guidelines [39–41]. The most effective treatment is to supplement estrogen. However, excessive estrogen can also increase the risk of uterine bleeding, as well as cardiovascular and other diseases in women [42, 43]. Traditional Chinese medicine formula JWYHD has become an ideal alternative therapy because of its good clinical effects and few side effects. Some studies have shown that aucubin can inhibit titanium particle-mediated apoptosis of MC3T3-E1 cells and promote osteogenesis by affecting the BMP2/Smads/RunX2 signal pathway [44]. Aucubin inhibited lipid accumulation and oxidative stress via the Nrf2/HO‐1 and AMPK signaling pathways [45]. Quercetin could promote the differentiation of bone marrow mesenchymal stem cells into osteoblasts and inhibit the differentiation of adipocytes into osteoblasts through Wnt/BMP and PPAR*γ* pathways [46]. Quercetin stimulates osteogenic differentiation of bone marrow stromal cells through the miRNA-206/connexin 43 pathway [47]. A naturally occurring naringenin derivative exerts potent bone anabolic effects by mimicking estrogen's action on osteoblasts [48]. Myricetin prevented alveolar bone loss in an experimental ovariectomized mouse model of periodontitis [49]. Myricetin and quercetin inhibited glucose uptake in isolated rat adipocytes [50].

All the experimental data showed that JWYHD could stimulate BMSCs to improve cell activity, osteogenic differentiation ability, and matrix mineralization ability; inhibit adipogenic differentiation; reduce bone metabolism and bone mass loss; and partially reverse the pathological process of PMOP induced by ovariectomy. Interestingly, MTT analysis of cell viability showed that with the increase of JWYHD concentration, the cell viability increased at first and then decreased. This phenomenon might be related to the stress effect of concentration-response. When the low concentration has a stimulating effect, the high concentration has an inhibitory effect. Various phenotypic assays in vivo showed that the functional disorder of BMSCs might be caused by the withdrawal effect of postmenopausal estrogen, which was characterized by the weakening of osteogenic ability, the enhancement of adipogenic ability, and the decrease in cell viability and proliferation in the process of BMSC differentiation.

**Figure 7:**
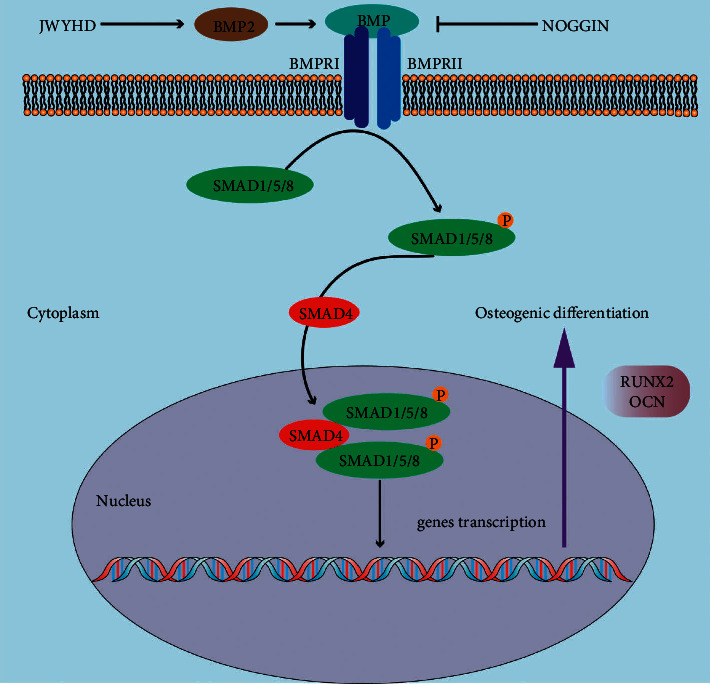
Mechanism of JWYHD activation of BMP signaling pathway and promotion of BMSC osteogenic differentiation. JWYHD activates BMP2 to bind to its receptor, leading to phosphorylation of SMAD1/5/8, while P-SMAD1/5/8 binds to SMAD4 and is transported to the nucleus to jointly regulate the transcription of downstream target genes RUNX2 and OCN.

There are many signal pathways to promote osteogenic differentiation of BMSCs, the most important of which is the BMP-SMAD signal pathway. BMP, or bone morphogenetic protein, is a member of the TGF-superfamily. BMP can stimulate DNA synthesis and cell replication, thus promoting the directional differentiation of mesenchymal cells into osteoblasts [51–53]. At the same time, it is also the main factor inducing bone and cartilage formation in vivo, especially for bone development and regeneration [54]. The binding of BMP and its receptor leads to the activation of Smad1/5/8 phosphorylation. Then, the phosphorylated Smad1/5/8 is combined with SMAD4 and transported to the nucleus, which jointly regulates the transcription of downstream target genes RUNX2 and OCN (Figure 7). Studies have shown that the deletion of BMP2 and P-Smad1/5/8 in BMSCs could inhibit the osteogenic differentiation induced by BMP in vitro and also show decreased osteogenic differentiation and bone mass loss in vivo, which was consistent with our data [55, 56]. RUNX2 is one of the best molecular pathways to regulate the bone formation, and it is necessary for BMSCs to transform into bone progenitor cells [57]. As a major transcriptional regulator of bone growth, RUNX2 is also involved in regulating OCN and COL1A1 and is expressed in both mouse and human bone progenitor cells [58]. Similarly, PPAR*γ* is a regulator of adipocyte production and differentiation, related to the pathology of many diseases, including obesity, diabetes, atherosclerosis, and cancer, and is also widely described as having anti-osteogenic effects [59]. They jointly regulate various cytokines to determine the choice of BMSC differentiation during osteogenesis and adipogenesis. In general, studies have found that osteogenic differentiation or adipogenic differentiation disorder of BMSCs may lead to osteoporosis, the ability of osteogenic differentiation is weakened, and the ability of adipogenic differentiation is enhanced accordingly, which restricts each other [60]. In addition, we found that PPAR*γ* as an adipogenic marker was upregulated in ovariectomized PMOP in vivo, and the same situation was found in vitro. Noggin, an inhibitor of the BMP pathway, downregulated osteogenesis-related genes, while PPAR*γ* was upregulated as an adipogenic marker. This study proved for the first time that JWYHD controlled the balance between osteogenic differentiation and adipogenic differentiation by activating the BMP-SMAD signal pathway and regulating BMSCs in vitro and in vivo.

In recent years, the coupling of bone formation and bone resorption by osteoblasts and osteoclasts has still been the focus of the treatment of osteoporosis [61]. The effect of regulating oxidative stress and autophagy on osteoporosis has also aroused widespread concern [62]. As a classic and particularly important pathway in regulating osteogenic differentiation of BMSCs, the BMP-SMAD signal has become a new hot spot and breakthrough in the treatment of osteoporosis. Previous studies have confirmed that JWYHD could prevent chondrocyte apoptosis, maintain the integrity of extracellular matrix, and reduce the inflammatory response, but there were still few studies on regulating the differentiation of stem cells or progenitor cells into osteoblasts, which makes our research particularly important.

All kinds of herbs in JWYHD play an important role, among which Radix Rehmanniae Praeparata accounts for 30%; Colla Cornus Cervi and papaya account for 15%; mustard seed accounts for 10%; Tetrandrine accounts for 6%; *Cinnamomum cassia*, Spatholobus stem, baked ginger, and licorice account for 5%; and ephedra accounts for 3%. Traditional Chinese medicine pursues syndrome differentiation and treatment, so the composition percentage of each herb is not fixed and can be adjusted according to the symptoms of the disease. JWYHD crude herbs (99 g) were decocted. The clinical dose was calculated according to the ratio of human to mouse (body weight 60 kg), the human dose was 1.65 g/kg, which was 0.165 g/100 g in rats. The concentration of the low-dose JWYHD group (L) was 1.0 g/ml, and the rats in the L group should be given 0.165 ml per 100 g of body weight of rats by gavage. The concentration of the middle-dose JWYHD group (M) was 1.5 g/ml, and the rats in group M were given 0.11 ml per 100 g of body weight of rats. The concentration of the high-dose JWYHD group (H) was 3.0 g/ml, and the rats in group H needed to be intragastrically administered 0.055 ml per 100 g of body weight.

This study bears some insufficiencies due to certain limitations. JWYHD regulates BMSCs to promote osteogenic differentiation, but there is a lack of flow cytometry data detection and identification, which hinders the consistency of results. MSCs extracted by normal steps have low purity and will contain some other cells. The positive rate of expression of BMSC surface markers CD105 and CD90 can be detected and identified by flow cytometry. Due to the lack of transmission electron microscope observation, the microstructure of BMSCs was not observed. In addition, the exact chemical composition of JWYHD has not been fully explored due to the complexity of TCM compound ingredients and targets.

## 5. Conclusion

In summary, in vitro and in vivo research data showed that JWYHD could regulate the balance between osteogenic differentiation and adipogenic differentiation of BMSCs by activating the BMP-SMAD signal pathway, improve bone metabolic parameters in ovariectomized PMOP rats, reduce bone mass loss, and partially reverse the pathological process of PMOP induced by ovariectomy. These findings provide sufficient evidence for JWYHD as a candidate drug for the treatment of PMOP.

## Figures and Tables

**Table 1:** Composition of Jiawei Yanghe decoction (JWYHD).

Herb name	English name	Latin name	Palace of origin	Weight (g)

Shu-Di-Huang	Rehmanniae Radix Praeparata	*Rehmannia glutinosa* (Gaertn.)	Henan, China	30
Rou-Gui	Cinnamomum cassia	*Cinnamomum cassia *presl	Yunnan, China	5
Ma-Huang	Ephedra	*Ephedra sinica* Stapf	Neimenggu, China	3
Ji-Xue-Teng	Suberect spatholobus stem	*Spatholobus suberectus* Dunn	Yunnan, China	5
Lu-Jiao-Jiao	Colla cornus cervi	Gelatinum cornu cervi	Jilin, China	15
Mu-Gua	Papaya	*Carica papaya* *L*	Guangdong, China	15
Fang-Ji	Tetrandrine	*Stephania tetrandra* S. Moore	Hubei, China	6
Bai-Jie-Zi	White mustard seed	Semen sinapis	Liaoning, China	10
Pao-Jiang	Baked ginger	*Zingiber officinale* Roscoe	Sichuan, China	5
Gan-Cao	Licorice	*Glycyrrhiza uralensis*	Xinjiang, China	5

**Table 2:** Quantitative analysis information of compounds in drug-containing serum.

No.	Compound	Formula	Molecular weight	RT (min)	Concentration (mM)

1	Aucubin	C15H22O9	346.332	16	0.083
2	Cinnamic aldehyde	C9H8O	132.16	1.979	0.052
3	Quercetin	C15H10O7	302.236	20.003	0.185
4	Naringenin	C15H12O5	272.25	14.65	0.047
5	Myricetin	C15H10O8	318.235	22.405	0.092
6	6-Gingerol	C17H26O4	294.391	9.317	0.019

## Data Availability

The datasets used or analyzed during the current study are available from the corresponding author on reasonable request.
